# Evaluation of Correlations of Flow Boiling Heat Transfer of R22 in Horizontal Channels

**DOI:** 10.1155/2013/458797

**Published:** 2013-07-17

**Authors:** Zhanru Zhou, Xiande Fang, Dingkun Li

**Affiliations:** Institute of Air Conditioning and Refrigeration, Nanjing University of Aeronautics and Astronautics, 29 Yudao Street, Nanjing 210016, China

## Abstract

The calculation of two-phase flow boiling heat transfer of R22 in channels is required in a variety of applications, such as chemical process cooling systems, refrigeration, and air conditioning. A number of correlations for flow boiling heat transfer in channels have been proposed. This work evaluates the existing correlations for flow boiling heat transfer coefficient with 1669 experimental data points of flow boiling heat transfer of R22 collected from 18 published papers. The top two correlations for R22 are those of Liu and Winterton (1991) and Fang (2013), with the mean absolute deviation of 32.7% and 32.8%, respectively. More studies should be carried out to develop better ones. Effects of channel dimension and vapor quality on heat transfer are analyzed, and the results provide valuable information for further research in the correlation of two-phase flow boiling heat transfer of R22 in channels.

## 1. Introduction

R22 is still widely used in chemical engineering and refrigeration and air-conditioning industries. Calculation of flow boiling heat transfer coefficients of R22 is important for design, development, and assessment of the systems and equipment using R22. A number of correlations for two-phase flow boiling heat transfer were proposed, and their applicability to R22 remains an issue. On the other hand, the study of R22 flow boiling heat transfer can provide useful information for revealing the influence of refrigerants on flow boiling heat transfer coefficients and for better understanding mechanisms of flow boiling heat transfer.

Greco and Vanoli [1] investigated experimentally flow boiling heat transfer coefficients of pure R22 and the azeotropic mixture R507 in a smooth horizontal stainless steel tube of 6 mm in inner diameter (ID) and 6 m in length, with the evaporating pressure ranging from 3 to 12 bar, mass fluxes from 250 kg/m^2^s to 286 kg/m^2^s, and heat fluxes from 10.6 kW/m^2^ to 17.0 kW/m^2^. They compared their measurements with the correlations of Shah [2], Kandlikar [3], Gungor and Winterton [4], Chen [5], and Yoshida et al. [6] and found that the correlations predicted the local heat transfer coefficient with good approximation at medium-low pressures (about 3.5 to 4.0 bar) but had strong underprediction at high pressures and remarkable overprediction at very low pressure.

Greco and Vanoli [7] measured flow boiling heat transfer coefficients of R22, R134a, R507, R404a, and R410a inside a smooth horizontal 6 mm ID tube with mass flux of about 36 kg/m^2^s, evaporating pressures ranging from 3 to 12 bar, and heat fluxes from 11 to 21 kW/m^2^. The experimental data are discussed in terms of the heat transfer coefficients as a function of the vapor quality. The experimental results indicated that, with increasing pressure, the nucleate-boiling contribution to the heat transfer coefficient increases mainly because of the corresponding decrease of the wall superheat required to form a stable nucleus.

Wang and Chiang [8] reported heat transfer characteristics for R22 and R407C in a 6.5 mm smooth tube at evaporation temperature of 2°C, mass fluxes from 100 to 400 kg/m^2^s, heat fluxes from 2.5 to 20 kW/m^2^, and vapor qualities form 0.1 to 0.89. They found that the heat transfer characteristics were dominated by nucleate boiling and that heat transfer coefficients of R407C were considerably lower than those of R22.

Shin et al. [9] examined experimentally flow boiling heat transfer of pure refrigerants (R22, R32, R134a, R290, and R600a) and refrigerant mixtures (R32/R134a, R290/R600a, and R32/R125) in a horizontal 7.7 mm ID stainless steel tube, obtaining the experimental points with heat fluxes from 10 to 30 kW/m^2^, mass fluxes from 424 to 742 kg/m^2^s, saturation temperatures at 12°C, and qualities from 0.06 to 0.84. The comparison of the measurements of R22 with the correlations of Gungor and Winterton [4, 27] showed that the Gungor and Winterton [4] correlation had the mean relative deviation (MRD) of −51.3% and the Gungor and Winterton [27] correlation had the MRD of −46.8%.

Oh and Son [10] investigated heat transfer coefficients of R22 and R134a evaporating in horizontal copper tubes with IDs of 1.77 mm, 3.36 mm, and 5.35 mm in the range of mass fluxes from 300 to 500 kg/m^2^s, heat fluxes of 10, 20, and 30 kW/m^2^, temperatures of 0 and 5°C, and qualities from 0.05 to 0.97. The measurements were analyzed and compared using seven previous heat transfer coefficient correlations proposed by Shah [2], Jung et al. [37], Gungor and Winterton [4], Liu and Winterton [24], Oh et al. [46], Wattelet et al. [26], and Yan and Lin [47]. The results showed that the Oh et al. [46] gave the best prediction, followed by the correlations of Liu and Winterton [24] and Wattelet et al. [26]. 

Choi et al. [11] conducted experiments of flow boiling heat transfer coefficients of R22, R32, R134a, R32/R134a. and R407C in a 7.75 mm ID horizontal smooth tube with mass fluxes from 240 to 640 kg/m^2^s, heat fluxes from 10.4 to 27.9 kW/m^2^s, saturation temperatures from −7 to 15.8°C, and vapor qualities from 0.04 to 0.9. They compared their measurements of pure refrigerants with the correlations of Jung et al. [37], Gungor and Winterton [4, 27], Kandlikar [3], and their own. It was shown that their own correlation had the smallest MAD of 13.2%, followed by the Kandlikar [3] correlation of 17.6%, the Gungor and Winterton [27] correlation of 26.4%, the Jung et al. [37] correlation of 33.2%, and the Gungor and Winterton [4] correlation of 34.5%.

Choi et al. [12] examined experimentally convective boiling heat transfer of R22, R134a, and CO_2_ in horizontal stainless steel tubes with IDs  of  1.5 mm and 3.0 mm, obtaining the local heat transfer coefficients with heat fluxes from 10 to 40 kW/m^2^, mass fluxes from 200 to 600 kg/m^2^s, a saturation temperature of 10°C, and quality up to 1.0. They believed that nucleate boiling heat transfer was the main contribution, particularly at the low quality region. They compared the measurements with the correlations of Wattelet et al. [26], Jung et al. [37], Kandlikar and Steinke [29], Tran et al. [38], Shah [2], Gungor and Winterton [27], and Chen [5]. It was shown that the Wattelet correlation had the smallest MAD of 19.1%, followed by the Jung et al. correlation of 23.5%, the Kandlikar-Steinke correlation of 24.3%, and the Tran et al. correlation of 24.8%. 

Wattelet et al. [13] conducted the experimental study of flow boiling heat transfer of R22 in 7.7 mm and 10.9 mm ID smooth, horizontal copper tubes with the parameter range of qualities from 0.12 to 0.96, mass fluxes from 49.6 to 512.9 kg/m^2^s, heat fluxes from 1.89 to 40 kW/m^2^, and saturation temperatures from −5.2 to 15.3°C. They selected three heat transfer correlations of Shah [2], Kandlikar [3], and Jung et al. [37] to compare with the experimental values. The results showed that the Kandlikar correlation had the smallest MAD of  13.5%, followed by the Jung et al. correlation of 18.9% and the Shah correlation of 20.2%.

Col [15] collected a new database during flow boiling of R22 in a horizontal 8 mm ID tube at a saturation temperature of 35°C, mass flux of 400 kg/m^2^s, and heat fluxes from 26.5 to 2.5 kW/m^2^. The comparison of the new database with the correlations of Gungor and Winterton [27], Liu and Winterton [24], Kandlikar [3], and Wojtan et al. [18] showed that the Gungor-Winterton correlation had the smallest MAD of 13.9%, followed by the Liu-Winterton correlation of 17.4% and the Wojtan et al. correlation of 21.7%.

Oh et al. [19] performed an experimental investigation of flow boiling heat transfer of R22, R134a, R410a, C_3_H_8_, and CO_2_ in horizontal 0.5 mm, 1.5 mm, and 3.0 mm ID stainless steel tubes. The experimental data ranges were heat fluxes from 5 to 40 kW/m^2^, mass fluxes from 50 to 600 kg/m^2^s, saturation temperatures from 0 to −15°C, and qualities up to 1.0. The measurements were compared with correlations of Gungor and Winterton [27], Jung et al. [37], Shah [2], Tran et al. [38], Chen [5], Wattelet et al. [26], Kandlikar [3], and Zhang [44]. It was shown that the Gungor and Winterton correlation had the smallest MAD 0f 25.8%, followed by the Jung et al. correlation of 26.8% and Shah correlation of 27.3%.

Jabardo and Filho [20] performed an experimental study of flow boiling of R22 in a 12.7 mm ID horizontal copper tube at evaporation temperatures 8 and 15°C, heat fluxes from 5 to 20 kW/m^2^, mass fluxes from 50 to 500 kg/m^2^s, and vapor qualities up to 1.0. They investigated effects of these physical parameters on the flow boiling heat transfer coefficients and evaluated two correlations of Jung and Radermacher [48] and Kandlikar [3] with the experimental data. The results showed that the Jung-Radermacher correlation was better than the Kandlikar correlation.

The previous brief review shows that most examinations of the correlations of flow boiling heat transfer coefficients of R22 were only based on the authors' own experimental data, and thus big differences existed among the evaluation results. In this paper, 1669 data points of flow boiling heat transfer of R22 are collected from 18 papers, and 26 existing flow boiling heat transfer correlations are evaluated based on the database. The evaluation results provide valuable information for developing new heat transfer prediction methods.

## 2. The Experimental Data for Flow Boiling Heat Transfer of R22

**Figure 1:**
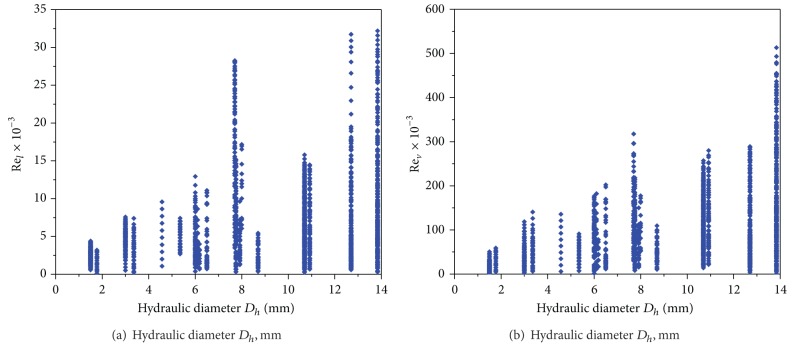
Distribution of channel size with Reynolds numbers diameter.

**Figure 2:**
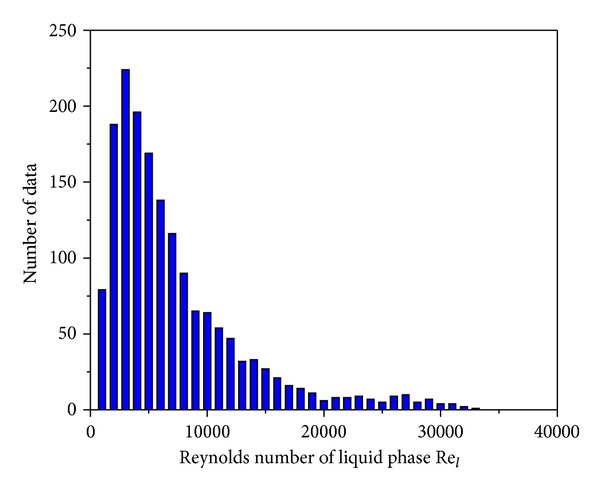
Liquid Reynolds number *Re*_*l*_ distribution.

**Figure 3:**
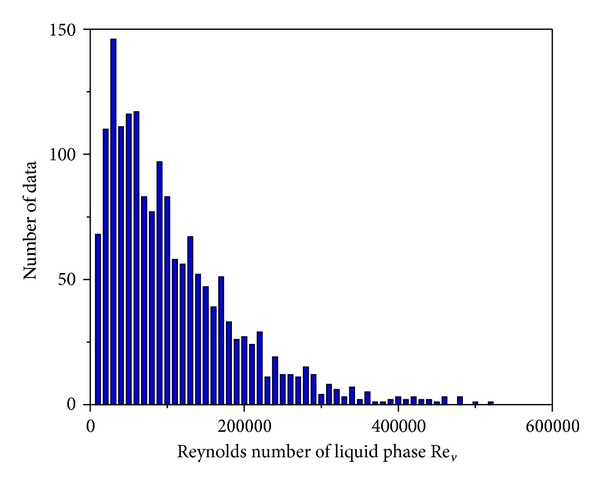
Vapor Reynolds number *Re*_*v*_ distribution.

From 18 published papers from 12 independent laboratories, 1669 data points of two-phase flow boiling heat transfer of R22 are obtained (Table 1). The experimental parameters varied in the following ranges: mass fluxes from 49.6 to 742 kg/m^2^s, heat fluxes from 1.9 to 57.5 kW/m^2^, vapor qualities from 0.006 to 0.982, saturation temperatures from −15.65 to 35°C, saturation pressures from 1.01 to 13.55 bar, and tube IDs from 1.5 to 13.84 mm. All the experiments were conducted with horizontal single circular smooth tubes.  Figure 1 demonstrates the distribution of the Reynolds number with the channel diameter. There are 157 data points in minichannel region (*D*_*h*_ < 3 mm) and other 1512 data points in conventional channel region (*D*_*h*_ ≥ 3 mm) according to the Kandlikar-Grande method [49].  Figure 2 shows the data points corresponding to the liquid Reynolds numbers ranging from 127.7 to 3.218  ×  10^4^ with most falling within 100–3  ×  10^4^.  Figure 3 illustrates the data points corresponding to the vapor Reynolds numbers ranging from 711.5 to 5.132  ×  10^5^ with most falling within 2000–5  ×  10^5^.

## 3. Correlations of Flow Boiling Heat Transfer Coefficients

With the database of the 1669 data points, 26 correlations of flow boiling heat transfer coefficients are evaluated, including those of Liu and Winterton [24], Fang [25], Shah [2], Kandlikar [3], Gungor and Winterton [4], Chen [5], Gungor and Winterton [27], Jung et al. [37], Wattelet et al. [26], Kandlikar and Steinke [29], Tran et al. [38], Zhang et al. [44], Lazarek and Black [31], Cooper [30], Kenning and Cooper [34], Kew and Cornwell [32], Warrier et al. [41], Yu et al. [45], Lee and Mudawar [42], Saitoh et al. [35], Bertsch et al. [28], Sun and Mishima [33], Hamdar et al. [40], Li and Wu [36, 43], and Kaew-On et al. [39].

The top 5 correlations which have an MAD ≤35% against the database (Table 1) are described briefly in the following. It is interesting that these correlations except for the Fang [25] correlation adopted and modified the Chen [5] additive method.

*(1)* *Liu-Winterton [24] Correlation*. Liu and Winterton [24] proposed the following correlation:

(1) $$\begin{array}{c} h_{\text{tp}}={\left[{\left(S\cdot h_{\text{nb}}\right)}^{2}+{\left(F\cdot h_{\text{sp},l}\right)}^{2}\right]}^{1/2}, \end{array}$$

where *h*_sp,*l*_ is the convective boiling heat transfer coefficient calculated by the Dittus and Boelter [50] correlation:

(2) $$\begin{array}{c} h_{\text{sp},l}=\frac{0.023\text{R}{\text{e}}_{l}^{0.8}\text{P}{\text{r}}_{l}^{0.4}\lambda_{l}}{D_{h}} \end{array}$$

and *h*_nb_ is the nucleate boiling heat transfer coefficient calculated by the following Cooper [30] correlation:

(3) $$\begin{array}{c} h_{\text{nb}}=55{P_{R}}^{0.12-0.087\ln\varepsilon }{\left(-0.4343\ln\;P_{R}\right)}^{-0.55}M^{-0.5}q^{0.67}, \end{array}$$

where **ε** is the surface roughness (*μ*m) and is set a value of 1 *μ*m if unknown, and the factors *S* and *F* are calculated by

(4) $$\begin{array}{c} F={\left[1+x\text{P}{\text{r}}_{l}\left(\frac{\rho_{l}}{\rho_{v}-1}\right)\right]}^{0.35},\\ S=\frac{1}{\left(1+0.055F^{0.1}\text{R}{\text{e}}_{l}^{0.16}\right)}. \end{array}$$

*(2)* *Fang [25] Correlation*. Fang [25] proposed the correlation from the CO_2_ database of 2956 experimental data points with a new dimensionless number Fa.

Consider

(5) $$\begin{array}{c} \text{Nu}=\frac{0.00061\left(S+F\right)\text{R}{\text{e}}_{l}\text{F}{\text{a}}^{0.11}\text{P}{\text{r}}_{l}^{0.4}}{\left[\ln\left(1.024\mu_{l,f}/\mu_{l,w}\right)\right]}\\ S=41000\text{B}{\text{o}}^{1.13}-0.275\\ F={\left(\frac{x}{1-x}\right)}^{a}{\left(\frac{\rho_{l}}{\rho_{v}}\right)}^{0.4}\\ a=\{\begin{array}{cc} 0.48+0.00524{\left(\text{R}{\text{e}}_{l}\text{F}{\text{a}}^{0.11}\right)}^{0.85} & \\ \;-5.9\times 10^{-6}{\left(\text{R}{\text{e}}_{l}\text{F}{\text{a}}^{0.11}\right)}^{1.85} & \text{R}{\text{e}}_{l}\text{F}{\text{a}}^{0.11}<600\\ 0.87 & 600\leq \text{R}{\text{e}}_{l}\text{F}{\text{a}}^{0.11}\leq 6000\\ \frac{160.8}{{\left(\text{R}{\text{e}}_{l}\text{F}{\text{a}}^{0.11}\right)}^{0.6}} & \text{R}{\text{e}}_{l}\text{F}{\text{a}}^{0.11}>6000. \end{array} \end{array}$$

*(3)* *Wattelet et al. [26] Correlation.* Wattelet et al. [26] proposed a correlation based on their own experimental data of R12, R134a, and a mixture flowing in 7.04 mm ID tube as follows:

(6) $$\begin{array}{c} h_{\text{tp}}={\left[h_{\text{nb}}^{2.5}+{\left(F\cdot R\cdot h_{\text{sp},l}\right)}^{2.5}\right]}^{1/2.5}, \end{array}$$

where *h*_sp,*l*_ is calculated with (2), *h*_np_ is calculated with (3), and the factors *F* and *R* are determined by

(7) $$\begin{array}{c} R=\{\begin{array}{cc} 1.32\text{F}{\text{r}}_{l}^{0.2} & \text{if}\;\text{F}{\text{r}}_{l}<0.25\\ 1 & \text{if}\;\text{F}{\text{r}}_{l}\geq 0.25 \end{array}\\ F=1+1.925X^{-0.83}, \end{array}$$

where *X* is the Lockhart-Martinelli parameter and is calculated by

(8) $$\begin{array}{c} X={\left(\frac{1-x}{x}\right)}^{0.9}{\left(\frac{\rho_{g}}{\rho_{l}}\right)}^{0.5}{\left(\frac{\mu_{l}}{\mu_{g}}\right)}^{0.1}. \end{array}$$

*(4)* *Gungor-Winterton [27] Correlation*. Gungor and Winterton [27] simplified their model [4] from the database of R11, R12, R22, R113, R114, and water and proposed

(9) $$\begin{array}{c} h_{\text{tp}}=\left(SS_{2}+FF_{2}\right)h_{\text{sp},l}, \end{array}$$

where *h*_sp,*l*_ is calculated with (2), and the factors *S, S*_2_ *, F, *and *F*_2_ are calculated by

(10) $$\begin{array}{c} S=1+3000\text{B}{\text{o}}^{0.86}\\ F=1.12{\left(\frac{x}{1-x}\right)}^{0.75}{\left(\frac{\rho_{l}}{\rho_{g}}\right)}^{0.41}\\ S_{2}=\{\begin{array}{cc} \text{F}{{\text{r}}_{l}}^{\left(0.1-2\text{F}{\text{r}}_{l}\right)} & \text{if}\;\text{horizontal}\;\text{and}\;\text{F}{\text{r}}_{l}<0.05\\ 1 & \text{otherwise} \end{array}\\ F_{2}=\{\begin{array}{cc} {\text{Fr}}_{l}^{1/2} & \text{if}\;\text{horizontal}\;\text{and}\;\text{F}{\text{r}}_{l}<0.05\\ 1 & \text{otherwise}. \end{array} \end{array}$$

*(5)* *Bertsch et al. [28] Correlation.* Bertsch et al. developed a composite correlation from a database of 12 different fluids based on the Chen [5] form.

Consider

(11) $$\begin{array}{c} h_{\text{tp}}=\left(1-x\right)h_{\text{nb}}+\left[1+80\left(x^{2}-x^{6}\right)e^{-0.6Co}\right]h_{\text{sp}}, \end{array}$$

where *h*_nb_ is calculated with (3)

(12) $$\begin{array}{c} h_{\text{sp}}=xh_{\text{sp},vo}+\left(1-x\right)h_{\text{sp},lo}\\ h_{\text{sp},ko}=\left[3.66+\frac{0.0668\text{R}{\text{e}}_{ko}\text{P}{\text{r}}_{k}D_{h}/L}{1+0.04{\left(\text{R}{\text{e}}_{ko}\text{P}{\text{r}}_{k}D_{h}/L\right)}^{2/3}}\right]\frac{\lambda_{k}}{D_{h}}\\ \text{R}{\text{e}}_{ko}=\frac{G_{\text{tp}}D_{h}}{\mu_{k}}, \end{array}$$

where *k* denotes either liquid or vapor.

## 4. Evaluation of Correlations of Flow Boiling Heat Transfer Coefficients for R22

With the database of the 1669 data points from 18 papers, 26 existing correlations of flow boiling heat transfer coefficients are assessed. The prediction deviations of the 26 correlations are listed in Table 2, where the MRD is the mean relative deviation and the MAD is the mean absolute deviation.

Consider

(13) $$\begin{array}{c} \text{MRD}=\frac{1}{N}\sum_{i=1}^{N}\frac{y{\left(i\right)}_{\text{pred}}-y{\left(i\right)}_{\exp}}{y{\left(i\right)}_{\exp}},\\ \text{MAD}=\frac{1}{N}\sum_{i=1}^{N}\left|\frac{y{\left(i\right)}_{\text{pred}}-y{\left(i\right)}_{\exp}}{y{\left(i\right)}_{\exp}}\right|. \end{array}$$

Table 2 lists the prediction deviations of the 26 correlations. 13 correlations have an MAD ≤40% against the database, which are Liu-Winterton [24], Fang [25], Wattelet et al. [26], Gungor-Winterton [27], Bertsch et al. [28], Kandlikar-Steinke [29], Kandlikar [3], Shah [2], Cooper [30], Gungor-Winterton [4], Lazarek-Black [31], Kew-Cornwell [32], and Sun-Mishima [33] correlations, with the MAD of 32.7%, 32.8%, 34.2%, 34.7%, 34.9%, 35.3%, 35.3%, 35.7%, 36.1%, 36.7%, 36.7%, 38.5%, and 39.4% and the MRD of −6.3%, 27.8%, 12.2%, 4.8%, −26%, 17.6%, 3.5%, −6%, 5.5%, −12.4%, 0.9%, and 24.4%, respectively. 13 correlations have an MAD >40% against the database, which are Kenning-Cooper [34], Saitoh et al. [35], Li-Wu [36], Jung et al. [37], Tran et al. [38], Kaew-On et al. [39], Hamdar et al. [40], Warrier et al. [41], Lee-Mudawar [42], Li and Wu [43], Zhang et al. [44], Chen [5], and Yu et al. [45] correlations.

Channel dimensions have effects on flow boiling heat transfer. A number of channel transition criteria were proposed. Kandlikar-Grande [49] proposed a method of defining *D*_*h*_ ≥ 3 mm as conventional channels, 200 *μ*m ≤*D*_*h*_ < 3 mm as minichannels, and 10 *μ*m ≤ *D*_*h*_ < 200 *μ*m as microchannels. Kew and Cornwell [32], Cheng et al. [51], Ong and Thome [52], and Fang et al. [53] classified two-phase heat transfer in channels according to the bond number Bd. They set different bond number as the threshold between minichannels and macrochannels. Another approach is the multidimensionless parameter method which combines at least two dimensionless parameters to form a criterion. Li and Wu [36] proposed Bd · *Re*_*l*_^0.5^ = 200 as the conventional-to-micro/minichannel criterion. Harirchian and Garimella [54] used the hybrid parameter Bd^0.5^ · Re_*l*o_ to classify channel dimensions.

**Figure 4:**
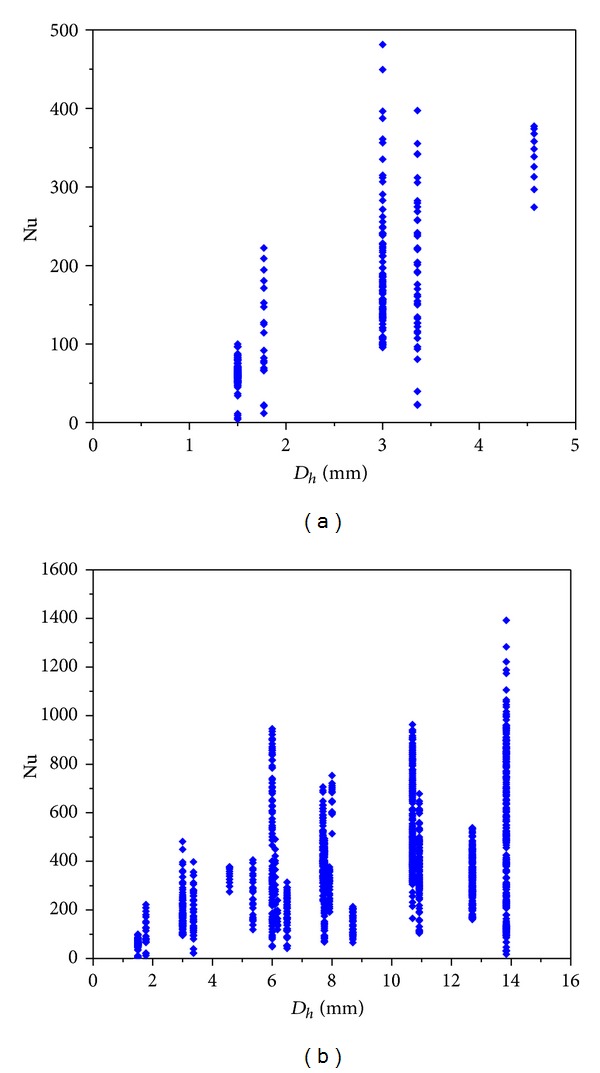
Statistics of the database with *D*_*h*_ and Nu as variables.

**Figure 5:**
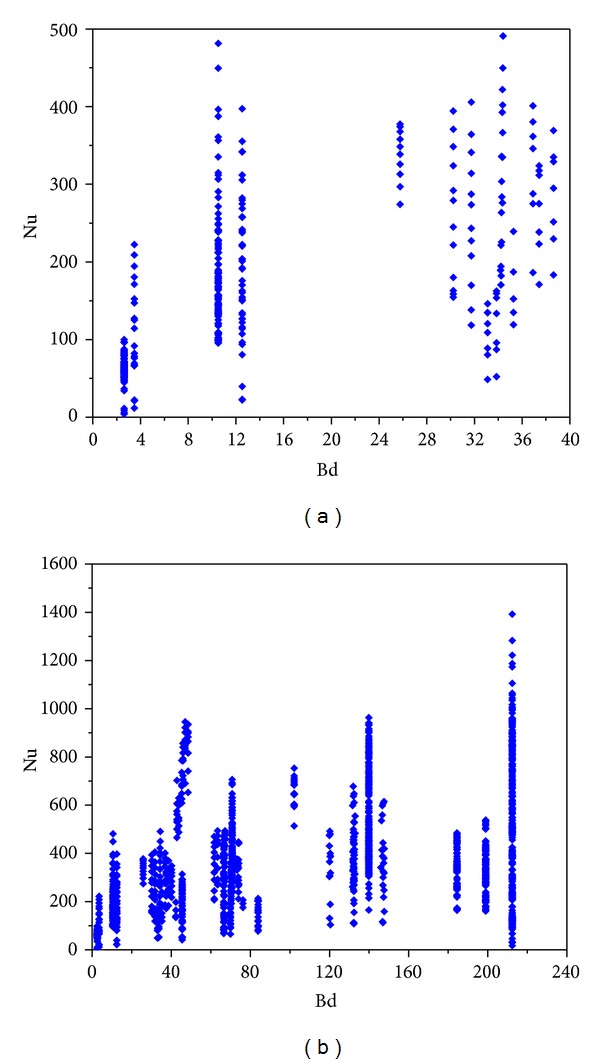
Statistics of the database with Bd and Nu as variables.

**Figure 6:**
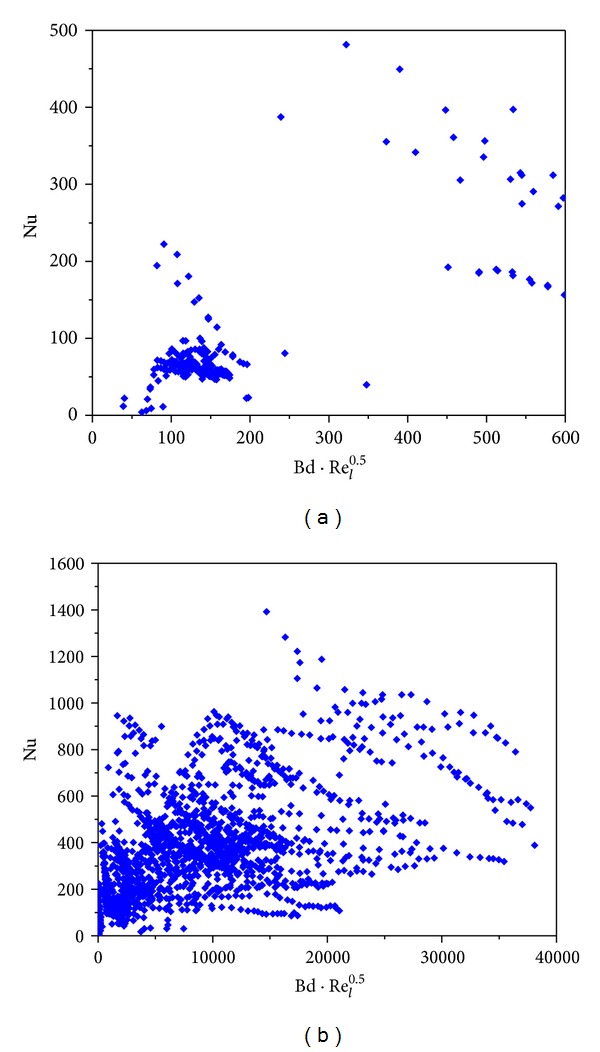
Statistics of the database with Bd · *Re*_*l*_^0.5^ and Nu as variables.

**Figure 7:**
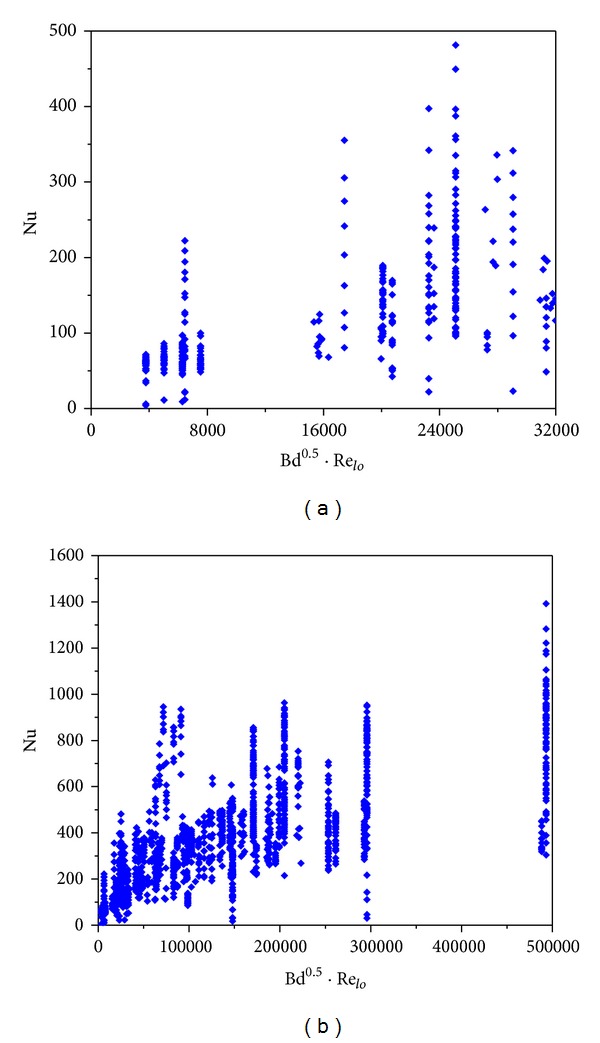
Statistics of the database with Bd^0.5^ · *Re*_*lo*_ and Nu as variables.

The variations of the Nu number with *D*_*h*_, Bd, Bd · *Re*_*l*_^0.5^, and Bd^0.5^ · Re_*lo*_ are shown in Figures 4–7.  Figure 4 demonstrates the variation of the Nu number with the channel diameter *D*_*h*_, which shows that it seems there is a transition taking place around *D*_*h*_ = 3 mm.  Figure 5 illustrates the distribution of the Nu number with the Bd number. No data were between Bd = 4 and Bd = 10, and thus it is insufficient to judge if there is a transition at Bd = 4. From Figure 6, it seems that there is a transition around Bd · *Re*_*l*_^0.5^ = 200, which coincides with the Li and Wu [36] result.  Figure 7 depicts the Nu number corresponding to the hybrid parameter Bd^0.5^ · *Re*_*lo*_. There are no data with Bd^0.5^ · *Re*_*lo*_ < 160, and thus the Harirchian and Garimella [54] conclusion cannot be verified. It is interesting to note that, in the R22 database, when *D*_*h*_ < 3 mm, Bd < 4 and when *D*_*h*_ ≥ 3 mm, Bd ≥ 4. Thus, the number of data in minichannel region with the Kandlikar-Grande method [49] is the same as that with the Kew-Cornwell method [32].

Table 3 shows the deviation of the top 5 correlations for different channel dimensions by the Kandlikar-Grande method and the Kew and Cornwell method. From the table, it can be seen that all the correlations perform better for conventional channels than for minichannels and that the Gungor-Winterton [27] correlation has the smallest MAD of 30.2% for conventional channels, while the Liu-Winterton [24] correlation has the smallest MAD of 40.1% for minichannels.

Table 4 demonstrates the deviation of the top 5 correlations for different channel dimensions by the Li-Wu method. It can be seen that all the correlations perform better for conventional channels than for minichannels. The Gungor-Winterton [27] correlation has the smallest MAD of 29.1% for conventional channels, while the Bertsch et al. [28] correlation has the smallest MAD of 40.1% for minichannels. Tables 3 and 4 show that all the top 5 correlations perform better for conventional channels than for minichannels and that the Gungor-Winterton [27] correlation has the smallest MAD for conventional channels in the three methods used in this work. 

**Figure 8:**
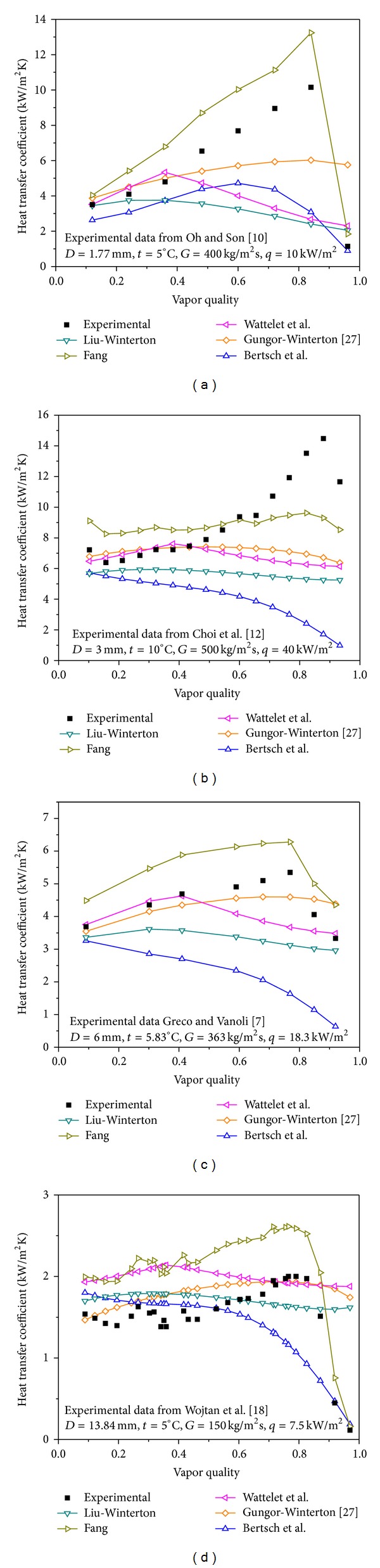
Effect of vapor quality on flow boiling heat transfer of R22: predicted versus measured.

Figure 8 demonstrates the trends of variation of heat transfer coefficient of the top 5 correlations with vapor quality. The experimental data used are from the Oh and Son [10] 1.77 mm ID tube in Figure 8(a), from the Choi et al. [12] 3 mm ID tube in Figure 8(b), from the Greco and Vanoli [7] 6 mm ID tube in Figure 8(c), and from the Wojtan et al. [18] 13.94 mm ID tube in Figure 8(d).  Table 5 illustrates the deviation of the top 5 correlations for different quality bands.

From Figure 8, the following can be seen.

1. For two-phase flow boiling of R22, the variation of heat transfer coefficients with vapor qualities is different for different channel dimensions. For the 1.77 mm tube (Figure 8(a)), the heat transfer coefficient increases quickly as the vapor quality increases continuously from low vapor quality and drops sharply at high vapor quality. The phenomenon of diving is caused by dry-out effects. For the 3 mm tube (Figure 8(b)), the heat transfer coefficient varies stably as the vapor quality increases from low values, increases rapidly with the vapor quality increasing, and then drops at higher vapor quality. For the 6 mm tube (Figure 8(c)), the heat transfer increases slowly with the quality increasing and decreases at high vapor quality. For the 13.84 mm tube (Figure 8(d)), the heat transfer coefficient keeps steady with the quality increasing at low values and drops at high quality. Figure 8 indicates that dry-out phenomena are more serious for minichannels than for conventional channels.
2. Figure 8 shows that none of the top 5 correlations has satisfactory ability to trace the trend of variation of heat transfer coefficient with quality. The Fang [25] correlation has certain ability to trace the trend of variation of heat transfer coefficient with quality, but it often overpredicts the experimental data. The Gungor-Winterton [27] correlation has some ability to trace the trend of variation of heat transfer coefficient with quality, but it has much smaller change rates when the dry-out phenomenon occurs.

From Table 5, the following can be seen that, in the range of 0 < *x* ≤ 0.3, the Bertsch et al. [28] correlation underpredicts the database, and the Liu-Winterton correlation has the smallest MAD of 20.2%, that, in the range of 0.3 < *x* ≤ 0.7, except for the Fang [25] and the Wattelet et al. [26] correlations, all under-predict the database, and the Gungor-Winterton [27] correlation has the smallest MAD of 23.5%, and that in the range of 0.7 < *x* < 1, except for the Bertsch et al. [28] correlation, all overpredict the database, and the Fang [25] correlation has the smallest MAD of 34%. The Liu-Winterton [24] and Bertsch et al. [28] correlations have smaller MAD in the range of 0 < *x* ≤ 0.3 than in the range of 0.3 < *x* ≤ 0.7, while the Fang [25], Wattelet et al. [26], and Gungor-Winterton [27] correlations have larger MAD in the range of 0 < *x* ≤ 0.3 than in the range of 0.3 < *x* ≤ 0.7. All the top 5 correlations have the largest MAD in the range of 0.7 < *x* < 1, although the Fang [25] correlation predicts much better than other correlations, indicating the failure in predicting the effect of dry out on the heat transfer coefficients.

## 5. Conclusions

1. The 26 existing correlations for flow boiling heat transfer are evaluated against the 1669 data points of two-phase flow boiling heat transfer of R22 collected from 18 published papers from 12 independent laboratories with the statistical calculation method.
2. Among the 26 correlations, the top two correlations are those of Liu and Winterton and Fang, with the MADs of 32.7% and 32.8%, respectively. It indicates the existing two-phase flow boiling heat transfer correlations cannot predict the heat transfer coefficients of R22 well. Therefore, more researches are needed to better understand the mechanism of flow boiling heat transfer to obtain more accurate values of the heat transfer coefficients.
3. There are 6 correlations having the MAD ≤35%. Following the Liu-Winterton and the Fang correlations are those of Wattelet et al., Gungor and Winterton [27], and Bertsch et al.
4. The statistics show that channel dimension has important effects on two-phase flow heat transfer of R22. This work characterizes channel dimensions with the channel size method, the Bd-type method, and the multidimensionless parameter method. Using these methods, all the top 5 correlations perform better for conventional channels than for minichannels. More investigations should be made to obtain better channel transition criterion to characterize channel dimensions.
5. The two-phase flow boiling heat transfer coefficients of R22 vary with vapor qualities. All the top 5 correlations have the worst prediction in the range of 0.7 to 1.0 because of dry-out phenomena, indicating that no correlation can predict the dry-out region. The dry-out effects on flow boiling heat transfer of R22 play an important role in developing a better correlation, and thus more research efforts should be made to understand dry-out mechanism for two-phase flow boiling heat transfer of R22.
6. A number of the correlations do not work well for R22, which may be because they were not developed for R22. For example, the Fang correlation developed from the CO_2_ database of 2956 experimental data points has an MAD of 15.5% for that database. However, its MAD for the R22 database of the present work reaches 32.8%.

## Figures and Tables

**Table 1:** Experimental data sources of flow boiling heat transfer coefficients of R22.

Data source	Parameter range: *T*_sat⁡_ (°C)/*p*_sat⁡_ (bar)/*G* (kg/m^2^s)/*q* (kW/m^2^)/*x *	Geometry range: *D*_*h*_ (mm)/*L* (mm)/Tube materials	Number of data points
Greco and Vanoli [7]	−3.2–18/1.01–8.6/363/18.3–21.8/0.01–0.97	6/6000/stainless steel	39
Greco and Vanoli [1]	−15.65–20/2.9–9.1/280/10.6–17/0.04–0.88	6/6000/stainless steel	50
Wang and Chiang [8]	2/5.3/100–400/2.5–20/0.1–0.89	6.5/1300/^a^	53
Shin et al. [9]	12/7.2/424–742/10–30/0.06–0.84	7.7/5900/stainless steel	85
Oh and Son [10]	0–5/4.98–5.84/300–500/10–30/0.05–0.97	*①* 1.77/160/; *②* 3.36/200/; *③* 5.35/260/Copper	88
Choi et al. [11]	−2.9–5.9/4.5–6/427/20.9/0.04–0.77	7.75/5900/Stainless steel	35
Choi et al. [12]	10/6.8/10–40/300–600/0.04–0.93	*①* 1.5/2000/; *②* 3/2000/Stainless steel	133
Wattelet et al. [13]	−5.22–15.28/4.19–7.96/ 49.6–512.9/1.89–40/0.12–0.96	*①* 7.747/1219.2/; *②* 10.922/1219.2/ copper	167
Park and Hrnjak [14]	−15/2.9/194.7–402.6/10/0.09–0.81	6.1/150/brass	18
Col [15]	35/13.5/400/26.5–52.5/0.21–0.72	8/1300/copper-constantan	15
Lallemand et al. [16]	14.148/7.7/150–300/10–30/0.08–0.97	10.7/500/^a^	288
Seo and Kim [17]	−15–5/2.9–5.8/70–164/5–15/0.16–0.85	*①* 8.7/3000; *②* 6.18/3000/^a^	52
Wojtan et al. [18]	5/5.84/100–500/2.1–57.5/0–0.98	13.84/2026/stainless steel	273
Oh et al. [19]	10/6.8/10–20/300–600/0.02–0.79	*①* 1.5/2000/; *②* 3/2000/Stainless steel	110
Jabardo and Filho [20]	8–15/6.4–7.9/50–500/5–20/0.04–0.93	12.7/1500/Copper	213
Oh et al. [21]	5/5.84/300/20/0.14–0.66	7.75/5000/stainless steel	10
Oh and Son [22]	15/7.9/400/20/0.03–0.89	4.57/4200/stainless steel	10
Kuo and Wang [23]	6–10/5.8–6.0/100–300/6–14/0.13–0.82	7.92/1300/^a^	30

^a^Tube material was not given.

**(a):** 

Deviation %	Liu and Winterton [24]	Fang [25]	Wattelet et al. [26]	Gungor and Winterton [27]	Bertsch et al. [28]	Kandlikar and Steinke [29]	Kandlikar [3]	Shah [2]	Cooper [30]	Gungor and Winterton [4]	Lazarek and Black [31]	Kew and Cornwell [32]	Sun and Mishima [33]
MRD	−6.3	27.8	12.2	4.8	−26.0	17.6	18.5	3.5	−6.0	5.5	−12.4	0.9	24.4
MAD	32.7	32.8	34.2	34.7	34.9	35.3	35.3	35.7	36.1	36.7	36.7	38.5	39.4

**Table:** (b)

Deviation %	Kenning and Cooper [34]	Saitoh et al. [35]	Li and Wu [36]	Jung et al. [37]	Tran et al. [38]	Kaew-On et al. [39]	Hamdar et al. [40]	Warrier et al. [41]	Lee and Mudawar [42]	Li and Wu [43]	Zhang et al. [44]	Chen [5]	Yu et al. [45]
MRD	3.0	5.4	−0.4	29.7	30.3	−44.8	−41.6	−65.9	−78.8	105.0	101.8	113.4	212.6
MAD	44.3	45.2	45.3	45.8	53.2	58.9	60.2	69.6	81.0	109.6	121.4	126.7	213.2

**Table 3:** Deviation of the top 5 correlations for different channel dimensions by Kandlikar and Grande method and by Kew and Cornwell method (%).

Data source	Channel type	Liu and Winterton [24]		Fang [25]		Wattelet et al. [26]		Gungor and Winterton [27]		Bertsch et al. [28]	
		MRD	MAD	MRD	MAD	MRD	MAD	MRD	MAD	MRD	MAD
Greco and Vanoli [7]	Macro^a^	−52.8	53.4	0.3	16.6	−43.4	44.9	−37.4	40.9	−69.4	69.4
Greco and Vanoli [1]	Macro	−26.1	54.8	15.9	35.5	−16.4	51.3	−3.0	60.8	−47.3	58.9
Wang and Chiang [8]	Macro	−2.2	20.7	58.8	58.8	12.7	25.5	23.4	28.4	−11.8	27.5
Shin et al. [9]	Macro	−5.8	17.9	29.0	29.6	14.7	22.8	9.2	15.7	−31.2	31.8
Oh and Son [10]	Mini^b^	8.0	66.5	28.4	29.9	25.3	70.6	81.6	108.1	−16.7	36.7
	Macro	16.5	51.5	19.8	23.9	37.3	58.7	43.3	60.5	2.2	31.3
Oh and Son [22]	Macro	−40.3	40.3	5.2	8.1	−27.3	27.3	−28.3	28.3	−53.9	53.9
Choi et al. [11]	Macro	−15.6	25.3	20.5	20.5	−0.1	24.3	3.8	15.9	−36.2	38.5
Choi et al. [12]	Mini	−0.8	18.6	43.6	43.8	22.4	26.9	27.2	28.4	−28.7	29.7
	Macro	−15.6	25.8	19.2	23.6	1.7	21.9	5.8	20.4	−31.1	35.4
Wattelet et al. [13]	Macro	−20.9	23.0	37.4	38.5	−5.3	17.8	−11.1	16.2	−29.2	32.9
Park and Hrnjak [14]	Macro	−44.1	44.1	21.8	21.9	−41.0	41.0	−22.6	22.6	−38.3	38.3
Col [15]	Macro	13.8	19.6	7.4	18.4	44.0	44.0	−12.7	13.6	−10.0	16.5
Lallemand et al. [16]	Macro	−29.9	31.0	8.4	16.8	−13.9	16.8	−30.8	31.7	−35.3	35.3
Seo and Kim [17]	Macro	−1.2	21.2	45.1	45.1	11.2	26.7	0.5	21.2	−18.6	28.6
Wojtan et al. [18]	Macro	19.9	52.7	26.6	36.6	42.2	60.3	19.7	48.5	−25.7	37.7
Oh et al. [19]	Mini	44.6	52.1	59.4	59.5	76.9	77.7	114.9	115.0	14.6	50.7
	Macro	3.8	18.4	31.6	31.6	24.5	27.7	28.4	28.7	−12.4	22.1
Jabardo and Filho [20]	Macro	−2.8	14.1	38.8	41.5	17.4	24.7	−7.0	13.9	−19.4	22.7
Oh et al. [21]	Macro	−30.3	30.3	11.1	11.1	−15.2	15.2	−21.8	21.8	−44.0	44.0
Kuo and Wang [23]	Macro	−33.2	33.2	19.6	19.6	−20.2	20.5	−25.4	25.4	−38.7	38.7

Total data	Mini	21.1	40.1	48.9	49.2	47.7	55.7	74.4	78.3	−7.3	40.2
	Macro	−9.2	32.0	25.6	31.1	8.5	32.0	−2.4	30.2	−27.9	34.3

^a^Macrochannel or conventional channel (*D*_*h*_ ≥ 3 mm, Bd ≥ 4). ^b^Minichannel (*D*_*h*_ < 3 mm, Bd < 4).

**Table 4:** Deviation of the top 5 correlations for different channel dimensions by Li and Wu method (%).

Data source	Channel type	Liu and Winterton [24]		Fang [25]		Wattelet et al. [26]		Gungor and Winterton [27]		Bertsch et al. [28]	
		MRD	MAD	MRD	MAD	MRD	MAD	MRD	MAD	MRD	MAD
Greco and Vanoli [7]	Macro^a^	−52.8	53.4	0.3	16.6	−43.4	44.9	−37.4	40.9	−69.4	69.4
Greco and Vanoli [1]	Macro	−26.1	54.8	15.9	35.5	−16.4	51.3	−3.0	60.8	−47.3	58.9
Wang and Chiang [8]	Macro	−2.2	20.7	58.8	58.8	12.7	25.5	23.4	28.4	−11.8	27.5
Shin et al. [9]	Macro	−5.8	17.9	29.0	29.6	14.7	22.8	9.2	15.7	−31.2	31.8
Oh and Son [10]	Mini^b^	58.6	111.8	24.5	28.6	83.5	124.7	147.8	171.8	−12.4	36.1
	Macro	−0.1	35.9	20.8	24.1	18.2	40.3	20.1	37.8	1.3	31.3
Oh and Son [22]	Macro	−40.3	40.3	5.2	8.1	−27.3	27.3	−28.3	28.3	−53.9	53.9
Choi et al. [11]	Macro	−15.6	25.3	20.5	20.5	−0.1	24.3	3.8	15.9	−36.2	38.5
Choi et al. [12]	Mini	−0.8	18.6	43.6	43.8	22.4	26.9	27.2	28.4	−28.7	29.7
	Macro	−15.6	25.8	19.2	23.6	1.7	21.9	5.8	20.4	−31.1	35.4
Wattelet et al. [13]	Macro	−20.9	23.0	37.4	38.5	−5.3	17.8	−11.1	16.2	−29.2	32.9
Park and Hrnjak [14]	Macro	−44.1	44.1	21.8	21.9	−41.0	41.0	−22.6	22.6	−38.3	38.3
Col [15]	Macro	13.8	19.6	7.4	18.4	44.0	44.0	−12.7	13.6	−10.0	16.5
Lallemand et al. [16]	Macro	−29.9	31.0	8.4	16.8	−13.9	16.8	−30.8	31.7	−35.3	35.3
Seo and Kim [17]	Macro	−1.2	21.2	45.1	45.1	11.2	26.7	0.5	21.2	−18.6	28.6
Wojtan et al. [18]	Macro	19.9	52.7	26.6	36.6	42.2	60.3	19.7	48.5	−25.7	37.7
Oh et al. [19]	Mini	44.6	52.1	59.4	59.5	76.9	77.7	114.9	115.0	14.6	50.7
	Macro	3.8	18.4	31.6	31.6	24.5	27.7	28.4	28.7	−12.4	22.1
Jabardo and Filho [20]	Macro	−2.8	14.1	38.8	41.5	17.4	24.7	−7.0	13.9	−19.4	22.7
Oh et al. [21]	Macro	−30.3	30.3	11.1	11.1	−15.2	15.2	−21.8	21.8	−44.0	44.0
Kuo and Wang [23]	Macro	−33.2	33.2	19.6	19.6	−20.2	20.5	−25.4	25.4	−38.7	38.7

Total data	Mini	28.0	46.7	48.1	48.8	55.5	63.4	83.6	87.5	−6.9	40.1
	Macro	−10.0	31.3	25.7	31.2	7.6	31.1	−3.5	29.1	−28.0	34.3

^a^Conventional channel (Bd · *Re*_*l*_^0.5^ > 200). ^b^Minichannel (Bd · *Re*_*l*_^0.5^ ≤ 200).

**Table 5:** Deviation of the top 5 correlations for different quality bands (%).

Data source	*x*	Liu and Winterton [24]		Fang [25]		Wattelet et al. [26]		Gungor and Winterton [27]		Bertsch et al. [28]	
		MRD	MAD	MRD	MAD	MRD	MAD	MRD	MAD	MRD	MAD
Greco and Vanoli [7]	(^a^0, 0.3]^b^	−47.5	50.3	5.5	13.1	−40.0	45.2	−40.8	46.0	−51.0	51.0
	(0.3,0.7]	−52.7	52.7	3.8	14.5	−41.2	41.6	−37.6	37.6	−66.2	66.2
	(0.7, 1)^a^	−56.0	56.0	−6.9	21.3	−48.1	48.8	−35.0	41.9	−83.8	83.8

Greco and Vanoli [1]	(0,0.3]	2.7	69.5	12.4	27.4	14.1	67.5	10.8	72.6	−18.8	56.5
	(0.3,0.7]	−27.9	48.5	21.4	37.5	−16.8	45.3	−1.5	57.2	−48.6	52.4
	(0.7,1)	−51.6	51.6	9.1	39.8	−46.1	46.1	−19.4	55.8	−73.2	73.2

Wang and Chiang [8]	(0,0.3]	20.5	22.8	42.8	42.8	33.4	33.8	26.8	29.8	−0.7	15.6
	(0.3,0.7]	−1.8	14.2	61.9	61.9	16.6	20.4	21.3	27.6	−4.5	24.0
	(0.7,1)	−27.4	31.5	69.8	69.8	−17.5	26.9	24.1	28.5	−38.6	47.3

Shin et al. [9]	(0,0.3]	9.5	13.8	42.4	42.4	26.7	27.4	19.3	21.5	−14.8	16.0
	(0.3,0.7]	−17.6	19.1	19.3	19.4	7.9	17.4	2.7	8.5	−42.8	42.8
	(0.7,1)	−36.4	36.4	−1.4	5.1	−22.7	22.7	−17.4	17.4	−69.7	69.7

Oh and Son [10]	(0,0.3]	30.3	31.0	39.2	39.2	49.2	49.2	49.3	49.3	32.0	35.8
	(0.3,0.7]	−27.4	27.8	16.4	17.7	−10.5	21.0	−7.5	15.9	−19.3	20.4
	(0.7,1)	95.1	162.6	6.5	21.2	124.2	183.8	207.1	247.7	−14.8	57.6

Oh and Son [22]	(0,0.3]	−31.5	31.5	10.2	10.2	−20.5	20.5	−26.1	26.1	−33.3	33.3
	(0.3,0.7]	−39.7	39.7	8.5	8.5	−22.8	22.8	−26.7	26.7	−52.9	52.9
	(0.7,1)	−49.9	49.9	−4.2	5.5	−40.2	40.2	−32.7	32.7	−75.7	75.7

Choi et al. [11]	(0,0.3]	6.0	20.1	23.5	23.5	20.8	31.3	16.0	28.4	−9.4	15.6
	(0.3,0.7]	−25.1	25.1	19.9	19.9	−7.8	17.2	−1.3	7.5	−48.3	48.3
	(0.7,1)	−42.9	42.9	13.3	13.3	−32.9	32.9	−13.0	13.0	−68.7	68.7

Choi et al. [12]	(0,0.3]	9.9	18.6	29.1	29.3	23.6	27.4	28.9	30.5	−17.5	22.5
	(0.3,0.7]	−19.1	21.9	38.1	38.4	7.9	19.2	11.4	17.3	−35.6	36.5
	(0.7,1)	−56.3	56.3	-20.3	20.3	−48.5	48.5	−41.5	41.5	−78.1	78.1

Wattelet et al. [13]	(0,0.3]	−6.9	9.7	31.3	31.3	8.4	10.1	−7.6	12.8	−18.3	20.3
	(0.3,0.7]	−19.1	21.6	38.7	39.0	−1.1	14.9	−8.9	15.4	−23.3	29.0
	(0.7,1)	−47.2	47.2	42.3	47.7	−38.0	38.0	−23.3	23.3	−63.4	63.4

Park and Hrnjak [14]	(0,0.3]	−19.1	19.2	10.0	10.4	−15.3	15.3	−14.0	14.0	−33.5	33.5
	(0.3,0.7]	−50.2	50.2	28.7	28.7	−46.5	46.5	−23.9	23.9	−33.3	33.3
	(0.7,1)	−69.3	69.3	25.7	25.7	−68.3	68.3	−32.9	32.9	−55.5	55.5

Col [15]	(0,0.3]	5.1	12.5	36.7	36.7	32.5	32.5	−9.8	10.2	7.4	13.2
	(0.3,0.7]	11.9	17.8	2.0	13.2	42.1	42.1	−14.9	15.5	−14.4	17.6
	(0.7,1)	60.2	60.2	−21.4	21.4	99.9	99.9	2.0	2.0	−14.0	14.0

Lallemand et al. [16]	(0,0.3]	−22.2	22.2	21.6	21.6	−6.2	6.9	−27.5	27.5	−18.5	18.5
	(0.3,0.7]	−31.4	31.4	9.9	15.0	−14.0	14.6	−31.5	31.5	−28.2	28.2
	(0.7,1)	−32.5	36.2	−3.2	16.5	−19.0	27.6	−31.8	34.8	−59.4	59.4

Seo and Kim [17]	(0,0.3]	15.3	21.9	41.3	41.3	26.1	28.8	6.1	13.4	−1.4	28.0
	(0.3,0.7]	−0.7	18.2	48.6	48.6	12.8	24.3	2.4	22.4	−14.0	20.6
	(0.7,1)	−22.5	29.7	39.2	39.2	−11.7	31.3	−12.0	27.3	−53.1	53.1

Wojtan et al. [18]	(0,0.3]	9.5	22.8	36.5	36.8	22.4	25.2	0.3	15.7	−0.7	25.2
	(0.3,0.7]	−11.2	24.4	27.3	31.7	8.5	25.0	−10.0	19.0	−27.1	33.6
	(0.7,1)	81.3	124.8	17.6	45.0	115.5	148.5	85.9	124.7	−43.4	54.5

Oh et al. [19]	(0,0.3]	19.2	22.3	31.3	31.5	32.6	33.8	39.6	39.7	−9.0	16.8
	(0.3,0.7]	3.6	19.7	58.0	58.0	38.7	40.7	49.8	50.0	−16.0	29.8
	(0.7,1)	560.0	560.0	144.8	144.8	663.7	663.7	1138.1	1138.1	486.0	486.0

Jabardo and Filho [20]	(0,0.3]	0.4	5.9	43.8	43.8	16.8	17.5	−9.9	14.4	−7.6	12.0
	(0.3,0.7]	−0.5	13.1	43.0	43.2	23.8	26.8	−4.0	14.2	−11.3	15.8
	(0.7,1)	−10.0	24.2	26.6	36.1	7.4	28.4	−8.9	12.8	−45.5	45.5

Oh et al. [21]^c^	(0,0.3]	−25.9	25.9	15.3	15.3	−12.4	12.4	−22.2	22.2	−33.0	33.0
	(0.3,0.7]	−32.2	32.2	9.3	9.3	−16.4	16.4	−21.7	21.7	−48.8	48.8

Kuo and Wang [23]	(0,0.3]	−24.3	24.3	18.7	18.7	−13.1	13.1	−25.1	25.1	−29.1	29.1
	(0.3,0.7]	−34.5	34.5	18.9	18.9	−19.8	20.4	−27.0	27.0	−38.7	38.7
	(0.7,1)	−42.8	42.8	23.5	23.5	−32.9	32.9	−20.6	20.6	−54.1	54.1

Total data	(0,0.3]	3.1	20.2	32.3	32.9	17.7	25.0	6.7	26.2	−10.3	22.0
	(0.3,0.7]	−16.9	24.7	29.8	32.4	3.7	23.2	−5.7	23.5	−26.3	30.6
	(0.7,1)	5.9	69.1	17.1	34.0	25.0	73.1	27.2	72.9	−46.7	62.6

^a^The number in the bracket is not covered. ^b^The number is covered. ^c^Vapor quality < 0.66.

## Nomenclature

Bd: Bond number, *g*(*ρ*_*l*_ − *ρ*_*g*_)*D*_*h*_^2^/*σ*
Bo: Boiling number, *q*/(*h*_*lg*_ *G*_tp_)
Co: Confinement number, $\sqrt{\sigma /\left[g(\rho_{l}-\rho_{g})D_{h}^{2}\right]}$
*D*_*h*_: Hydraulic diameter (m)
Fa: Fang number, (*ρ*_*l*_ − *ρ*_*g*_)*σ*/(*G* ^2^ *D*_*h*_)
Fr: Froude number
*G*: Mass flux (kg/m^2^ · s)
*h*: Heat transfer coefficient (W/m^2^ · K)
*h*_*lv*_: Latent heat of vaporization (J kg^−1^)
*L*: Tube length (m)
*M*: Molecular mass (kg/kmol)
Nu: Nusselt number
*P*_*R*_: Reduced pressure, *p*/*p*_crit_
Pr: Prandtl number
*q*: Heat flux from tube wall to fluid (W/m^2^)
*Re*: Reynolds number
*t*: Temperature (°C)
*x*: Vapor quality
*X*: Lockhart-Martinelli parameter.

### Greek Symbols

*λ*: Thermal conductivity (W/m · K)
*ρ*: Density (kg/m^3^)
*σ*: Surface tension (N/m)
*ε*: Surface roughness (*μ*m), void fraction.

### Subscripts

exp⁡: Experimental
*f*: Fluid
*v*: Saturated vapor
*vo*: All flow taken as vapor
*l*: Saturated liquid
*lo*: All flow taken as liquid
nb: Nucleate boiling
pred: Predicted
sp: Single-phase
*t*: Turbulent
tp: Two-phase
*w*: Channel wall inner surface.

## References

[1] Greco A, Vanoli GP (2004). Evaporation of refrigerants in a smooth horizontal tube: prediction of R22 and R507 heat transfer coefficients and pressure drop. *Applied Thermal Engineering*.

[2] Shah MM (1982). Chart correlation for saturated boiling heat transfer: equations and further study. *ASHRAE Transactions*.

[3] Kandlikar SG (1990). General correlation for saturated two-phase flow boiling heat transfer inside horizontal and vertical tubes. *Journal of Heat Transfer*.

[4] Gungor KE, Winterton RHS (1986). A general correlation for flow boiling in tubes and annuli. *International Journal of Heat and Mass Transfer*.

[5] Chen JC (1963). A correlation for boiling heat transfer to saturated fluid in convective flow. *ASME Paper*.

[6] Yoshida S, Mari H, Hong H, Matsunaga T (1994). Prediction of binary mixture bowling heat transfer coefficient using only phase equilibrium data. *Transaction JAR*.

[7] Greco A, Vanoli GP (2005). Flow-boiling of R22, R134a, R507, R404A and R410A inside a smooth horizontal tube. *International Journal of Refrigeration*.

[8] Wang C, Chiang C (1997). Two-phase heat transfer characteristics for R-22/R-407C in a 6.5 mm smooth tube. *International Journal of Heat and Fluid Flow*.

[9] Shin JY, Kim MS, Ro ST (1997). Experimental study on forced convective boiling heat transfer of pure refrigerants and refrigerant mixtures in a horizontal tube. *International Journal of Refrigeration*.

[10] Oh H, Son C (2011). Evaporation flow pattern and heat transfer of R-22 and R-134a in small diameter tubes. *Heat and Mass Transfer*.

[11] Choi TY, Kim YJ, Kim MS, Ro ST (2000). Evaporation heat transfer of R-32, R-134a, R-32/134a, and R-32/125/134a inside a horizontal smooth tube. *International Journal of Heat and Mass Transfer*.

[12] Choi K, Pamitran AS, Oh C, Oh J (2007). Boiling heat transfer of R-22, R-134a, and CO2 in horizontal smooth minichannels. *International Journal of Refrigeration*.

[13] Wattelet JP, Chato JC, Christoffersen BR (1994). Heat transfer flow regimes of refrigerants in a horizontal-tube evaporator. *ACRC*.

[14] Park CY, Hrnjak PS (2007). CO2 and R410A flow boiling heat transfer, pressure drop, and flow pattern at low temperatures in a horizontal smooth tube. *International Journal of Refrigeration*.

[15] Col DD (2010). Flow boiling of halogenated refrigerants at high saturation temperature in a horizontal smooth tube. *Experimental Thermal and Fluid Science*.

[16] Lallemand M, Branescu C, Haberschill P (2001). Local heat transfer coefficients during boiling of R22 and R407C in horizontal smooth and microfin tubes. *International Journal of Refrigeration*.

[17] Seo K, Kim Y (2000). Evaporation heat transfer and pressure drop of R-22 in 7 and 9.52 mm smooth/micro-fin tubes. *International Journal of Heat and Mass Transfer*.

[18] Wojtan L, Ursenbacher T, Thome JR (2005). Investigation of flow boiling in horizontal tubes: part II-development of a new heat transfer model for stratified-wavy, dryout and mist flow regimes. *International Journal of Heat and Mass Transfer*.

[19] Oh J, Pamitran AS, Choi K, Hrnjak P (2011). Experimental investigation on two-phase flow boiling heat transfer of five refrigerants in horizontal small tubes of 0.5, 1.5 and 3.0 mm inner diameters. *International Journal of Heat and Mass Transfer*.

[20] Jabardo JMS, Filho EPB (2000). Convective boiling of halocarbon refrigerants flowing in a horizontal copper tube-an experimental study. *Experimental Thermal and Fluid Science*.

[21] Oh HK, Ku H-G, Roh G-S, Son C, Park S-J (2008). Flow boiling heat transfer characteristics of carbon dioxide in a horizontal tube. *Applied Thermal Engineering*.

[22] Oh HK, Son C-H (2011). Flow boiling heat transfer and pressure drop characteristics of CO2 in horizontal tube of 4.57 mm inner diameter. *Applied Thermal Engineering*.

[23] Kuo CS, Wang CC (1996). In-tube evaporation of HCFC-22 in a 9.52 mm micro-fin/smooth tube. *International Journal of Heat and Mass Transfer*.

[24] Liu Z, Winterton RHS (1991). A general correlation for saturated and subcooled flow boiling in tubes and annuli, based on a nucleate pool boiling equation. *International Journal of Heat and Mass Transfer*.

[25] Fang XD (2013). A new correlation of flow boiling heat transfer coefficients for carbon dioxide. *International Journal of Heat and Mass Transfer*.

[26] Wattelet JP, Chato JC, Souza AL, Christoffersen BR Evaporative characteristics of R-12, R-134a, and a mixture at low mass fluxes.

[27] Gungor KE, Winterton RHS (1987). Simplified general correlation for saturated flow boiling and comparison with data. *Chemical Engineering Research and Design*.

[28] Bertsch SS, Groll EA, Garimella SV (2009). A composite heat transfer correlation for saturated flow boiling in small channels. *International Journal of Heat and Mass Transfer*.

[29] Kandlikar SG, Steinke ME Predicting heat transfer during flow boiling in minichannels and microchannels.

[30] Cooper MG (1984). Heat flow rates in saturated nucleate pool boiling-a wide-ranging examination using reduced properties. *Advances in Heat Transfer C*.

[31] Lazarek GM, Black SH (1982). Evaporative heat transfer, pressure drop and critical heat flux in a small vertical tube with R-113. *International Journal of Heat and Mass Transfer*.

[32] Kew PA, Cornwell K (1997). Correlations for the prediction of boiling heat transfer in small-diameter channels. *Applied Thermal Engineering*.

[33] Sun L, Mishima K (2009). An evaluation of prediction methods for saturated flow boiling heat transfer in mini-channels. *International Journal of Heat and Mass Transfer*.

[34] Kenning DBR, Cooper MG (1989). Saturated flow boiling of water in vertical tubes. *International Journal of Heat and Mass Transfer*.

[35] Saitoh S, Daiguji H, Hihara E (2007). Correlation for boiling heat transfer of R-134a in horizontal tubes including effect of tube diameter. *International Journal of Heat and Mass Transfer*.

[36] Li W, Wu Z (2010). A general criterion for evaporative heat transfer in micro/mini-channels. *International Journal of Heat and Mass Transfer*.

[37] Jung DS, McLinden M, Radermacher R, Didion D (1989). A study of flow boiling heat transfer with refrigerant mixtures. *International Journal of Heat and Mass Transfer*.

[38] Tran TN, Wambsganss MW, France DM (1996). Small circular- and rectangular-channel boiling with two refrigerants. *International Journal of Multiphase Flow*.

[39] Kaew-On J, Sakamatapan K, Wongwises S (2011). Flow boiling heat transfer of R134a in the multiport minichannel heat exchangers. *Experimental Thermal and Fluid Science*.

[40] Hamdar M, Zoughaib A, Clodic D (2010). Flow boiling heat transfer and pressure drop of pure HFC-152a in a horizontal mini-channel. *International Journal of Refrigeration*.

[41] Warrier GR, Dhir VK, Momoda LA (2002). Heat transfer and pressure drop in narrow rectangular channels. *Experimental Thermal and Fluid Science*.

[42] Lee J, Mudawar I (2005). Two-phase flow in high-heat-flux micro-channel heat sink for refrigeration cooling applications: part II-heat transfer characteristics. *International Journal of Heat and Mass Transfer*.

[43] Li W, Wu Z (2010). A general correlation for evaporative heat transfer in micro/mini-channels. *International Journal of Heat and Mass Transfer*.

[44] Zhang W, Hibiki T, Mishima K (2004). Correlation for flow boiling heat transfer in mini-channels. *International Journal of Heat and Mass Transfer*.

[45] Yu W, France DM, Wambsganss MW, Hull JR (2002). Two-phase pressure drop, boiling heat transfer, and critical heat flux to water in a small-diameter horizontal tube. *International Journal of Multiphase Flow*.

[46] Oh HK, Katsuta M, Shibata K Heat transfer characteristics of R134a in a capillary tube heat exchanger.

[47] Yan YY, Lin TF (1998). Evaporation heat transfer and pressure drop of refrigerant R-134a in a small pipe. *International Journal of Heat and Mass Transfer*.

[48] Jung DS, Radermacher R (1991). Prediction of heat transfer coefficient of various refrigerants during evaporation. ASHRAE annual meeting. *Paper*.

[49] Kandlikar SG, Grande WJ (2003). Evolution of microchannel flow passages-thermohydraulic performance and fabrication technology. *Heat Transfer Engineering*.

[50] Dittus FW, Boelter LMK (1930). Heat transfer in automobile radiator of the tubular type. *University of California Publications in Engineering*.

[51] Cheng P, Wu H-Y, Hong F-J (2007). Phase-change heat transfer in microsystems. *Journal of Heat Transfer*.

[52] Ong CL, Thome JR (2011). Macro-to-microchannel transition in two-phase flow: part 1-two-phase flow patterns and film thickness measurements. *Experimental Thermal and Fluid Science*.

[53] Fang XD, Zhou ZR, Li DK (2013). Review of correlations of flow boiling heat transfer coefficients for carbon dioxide. *International Journal of Refrigeration*.

[54] Harirchian T, Garimella SV (2010). A comprehensive flow regime map for microchannel flow boiling with quantitative transition criteria. *International Journal of Heat and Mass Transfer*.

